# Predicting pediatric age from chest X-rays using deep learning: a novel approach

**DOI:** 10.1186/s13244-025-02068-5

**Published:** 2025-08-23

**Authors:** Maolin Li, Jiang Zhao, Huanhuan Liu, Biao Jin, Xuee Cui, Dengbin Wang

**Affiliations:** 1 Department of Radiology, Xinhua Hospital Affiliated to Shanghai Jiao Tong University School of Medicine, Shanghai, China; 2 Department of Radiology, Shanghai Tenth People’s Hospital, Tongji University, Shanghai, China

**Keywords:** Deep learning, Age prediction, Pediatric growth, Chest X-ray

## Abstract

**Objectives:**

Accurate age estimation is essential for assessing pediatric developmental stages and for forensics. Conventionally, pediatric age is clinically estimated by bone age through wrist X-rays. However, recent advances in deep learning enable other radiological modalities to serve as a promising complement. This study aims to explore the effectiveness of deep learning for pediatric age estimation using chest X-rays.

**Materials and methods:**

We developed a ResNet-based deep neural network model enhanced with Coordinate Attention mechanism to predict pediatric age from chest X-rays. A dataset comprising 128,008 images was retrospectively collected from two large tertiary hospitals in Shanghai. Mean Absolute Error (MAE) and Mean Absolute Percentage Error (MAPE) were employed as main evaluation metrics across age groups. Further analysis was conducted using Spearman correlation and heatmap visualizations.

**Results:**

The model achieved an MAE of 5.86 months for males and 5.80 months for females on the internal validation set. On the external test set, the MAE was 7.40 months for males and 7.29 months for females. The Spearman correlation coefficient was above 0.98, indicating a strong positive correlation between the predicted and true age. Heatmap analysis revealed the deep learning model mainly focused on the spine, mediastinum, heart and great vessels, with additional attention given to surrounding bones.

**Conclusions:**

We successfully constructed a large dataset of pediatric chest X-rays and developed a neural network model integrated with Coordinate Attention for age prediction. Experiments demonstrated the model’s robustness and proved that chest X-rays can be effectively utilized for accurate pediatric age estimation.

**Critical relevance statement:**

By integrating pediatric chest X-rays with age data using deep learning, we can provide more support for predicting children’s age, thereby aiding in the screening of abnormal growth and development in children.

**Key Points:**

- This study explores whether deep learning could leverage chest X-rays for pediatric age prediction.
- Trained on over 120,000 images, the model shows high accuracy on internal and external validation sets.
- This method provides a potential complement for traditional bone age assessment and could reduce radiation exposure.

**Graphical Abstract:**

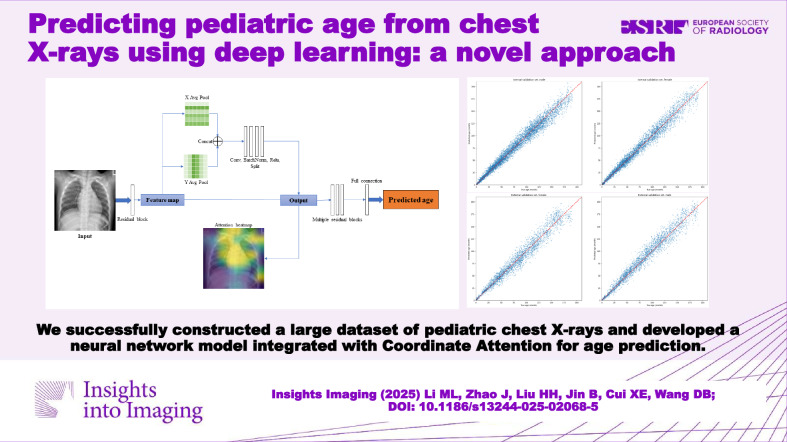

## Introduction

Predicting a child’s age is an important aspect of pediatric practice and is primarily used to assess growth and development. Pediatric development and growth assessments are critical components of child healthcare [1, 2], enabling the early identification and treatment of potential developmental disorders and underlying medical conditions [3]. Radiological evaluation plays a crucial role in assessing pediatric development, especially in predicting bone age, helping to diagnose abnormal growth and development in children, and guiding treatment. For example, in the setting of growth hormone deficiency or precocious puberty, wrist X-rays are the most important radiological method to assess a child’s bone age [4]. Besides, other skeletal radiographs, such as those of the clavicle [5, 6], knee joint [7], and pelvis [8], and even dental films [9–11] and brain Magnetic resonance imaging (MRI) [12], have also shown value in evaluating pediatric development age.

With the rapid development of deep learning in medical image analysis, age assessment algorithms based on deep neural networks have gradually reached high performance, even surpassing those of human experts [13–18]. Some of these algorithms do not rely on traditional methods; instead, they evaluate the entire image holistically [19], which involves integrating information by identifying features that were previously regarded as imperceptible to the human. This means that with deep learning, information can be extracted from medical images that cannot be observed by radiologists. Because it does not rely on prior medical knowledge of a human radiologist, non-wrist X-rays have the potential to be a reliable indicator of growth and development in children. This method has been applied to other body parts, such as knee joint X-ray [20] and abdominal CT scout views [21], and has demonstrated a certain level of value.

Chest X-ray is currently the most common radiological examination undertaken in pediatric practice [22, 23]. In clinical practice, chest X-ray is an important means for diagnosing respiratory diseases in children, and it also provides information about children’s growth and development. It would be a major step forward if the large number of chest X-rays available were available to assess pediatric age and growth. Although some studies have attempted to use chest X-rays to predict age, these have primarily focused on adults [21, 24–26]. Research specifically aimed at predicting age using pediatric chest X-rays has yet to be explored and validated.

Based on existing research, deep learning has great potential in predicting the age of chest X-rays. This study aims to implement a deep learning model to predict the age of children through chest X-rays.

## Materials and methods

### Data collection

We continuously collected pediatric chest X-ray data from two tertiary hospitals in China between 2021 and 2024. The dataset from Xinhua Hospital (Hospital A) was split into training (90%) and internal validation (10%) sets for the deep learning model. Meanwhile, the entire dataset from Tenth People’s Hospital (Hospital B) was used solely for external validation. Additionally, to account for differences in growth and development between genders, the data for males and females were separated for model training and validation.

The inclusion criteria were limited to normal chest X-rays of Chinese children aged 0 to 16 years for both hospitals. Standing, supine, and bedside chest X-rays obtained from multiple machines with diverse scanning parameters were all included in this study. Exclusion criteria were based on three primary factors: clinical history of growth disorders, positive findings, and poor image quality: (1) Patients with a history of growth or developmental disorder were excluded after reviewing their patient histories, including diagnoses such as “developmental disorders,” “precocious puberty,” “dwarfism,” or any other indications of abnormal growth. (2) Cases with obvious positive findings on chest X-rays, including pneumonia, atelectasis, pneumothorax, pulmonary nodules, cardiac or mediastinum abnormalities, or chest wall or spinal deformities, etc., were excluded. (3) Low-quality images were excluded from the dataset, including unclear bedside chest X-rays, suboptimal positioning due to poor patient cooperation, the presence of foreign objects, and issues with over- or under-exposure. The exclusion of growth disorder was intended to ensure model stability during the initial development phase. Children with growth or developmental disorders may present physiological features inconsistent with their chronological age, leading the model to learn mismatched image-age patterns. Therefore, our dataset was intentionally restricted to normally developing children to establish a reliable baseline model.

**Fig. 1:**
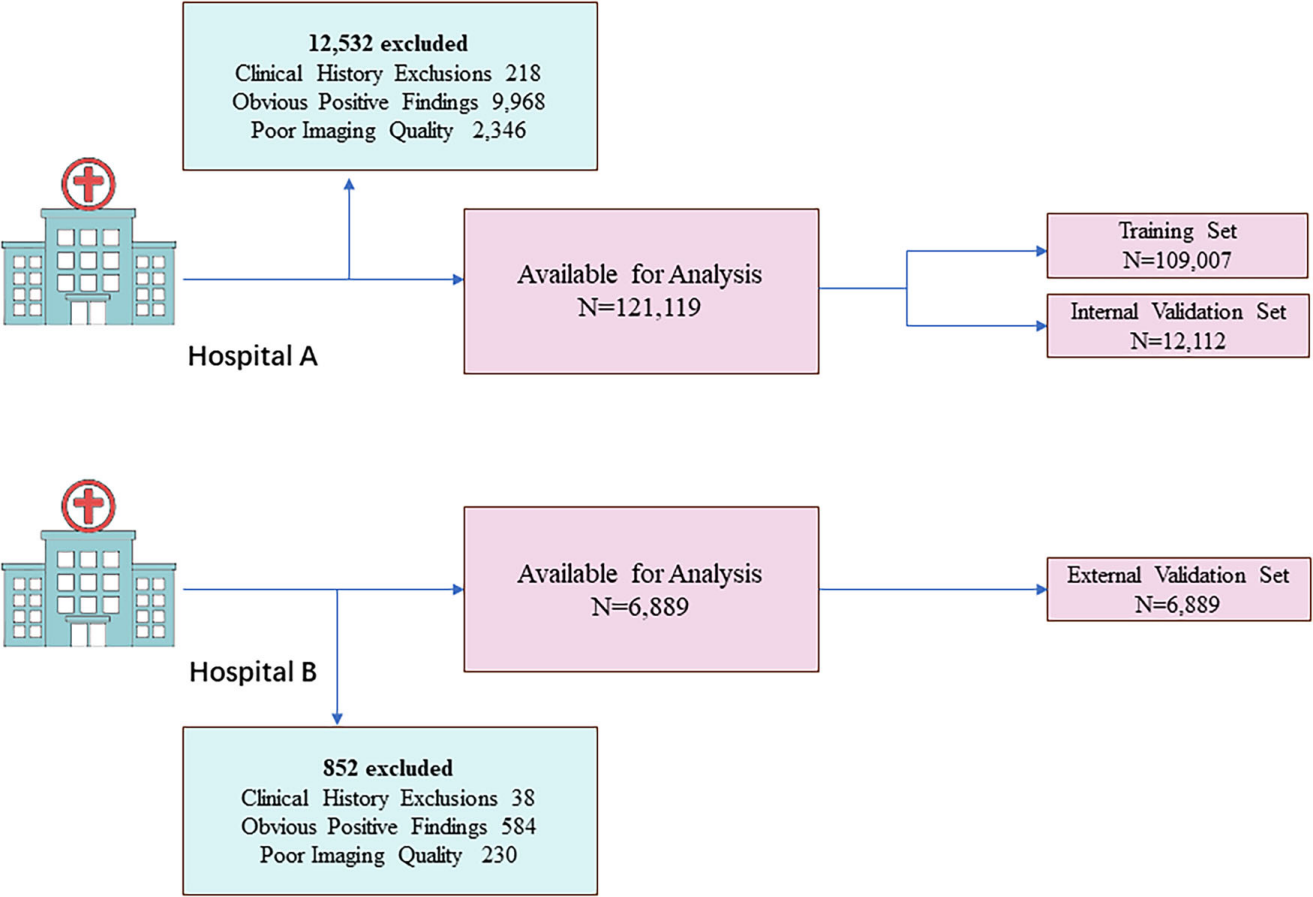
Data collection and split process

Figure 1 illustrates the entire process of data collection, exclusion, and dataset split.

This study was reviewed and approved by the IRB of Xinhua Hospital affiliated to Shanghai Jiao Tong University School of Medicine with approval number: XHEC-D-2024-143, dated 2024/8/28, and by the IRB of Shanghai Tenth People’s Hospital with approval number: SHSY-IEC-5.0/24K135/P01, dated 2024/9/11. Patient informed consent was exempted by the institution’s ethical review board. All data used in this study were completely anonymized and did not involve any personal privacy or commercial interests.

### Neural network implementation

**Fig. 2:**
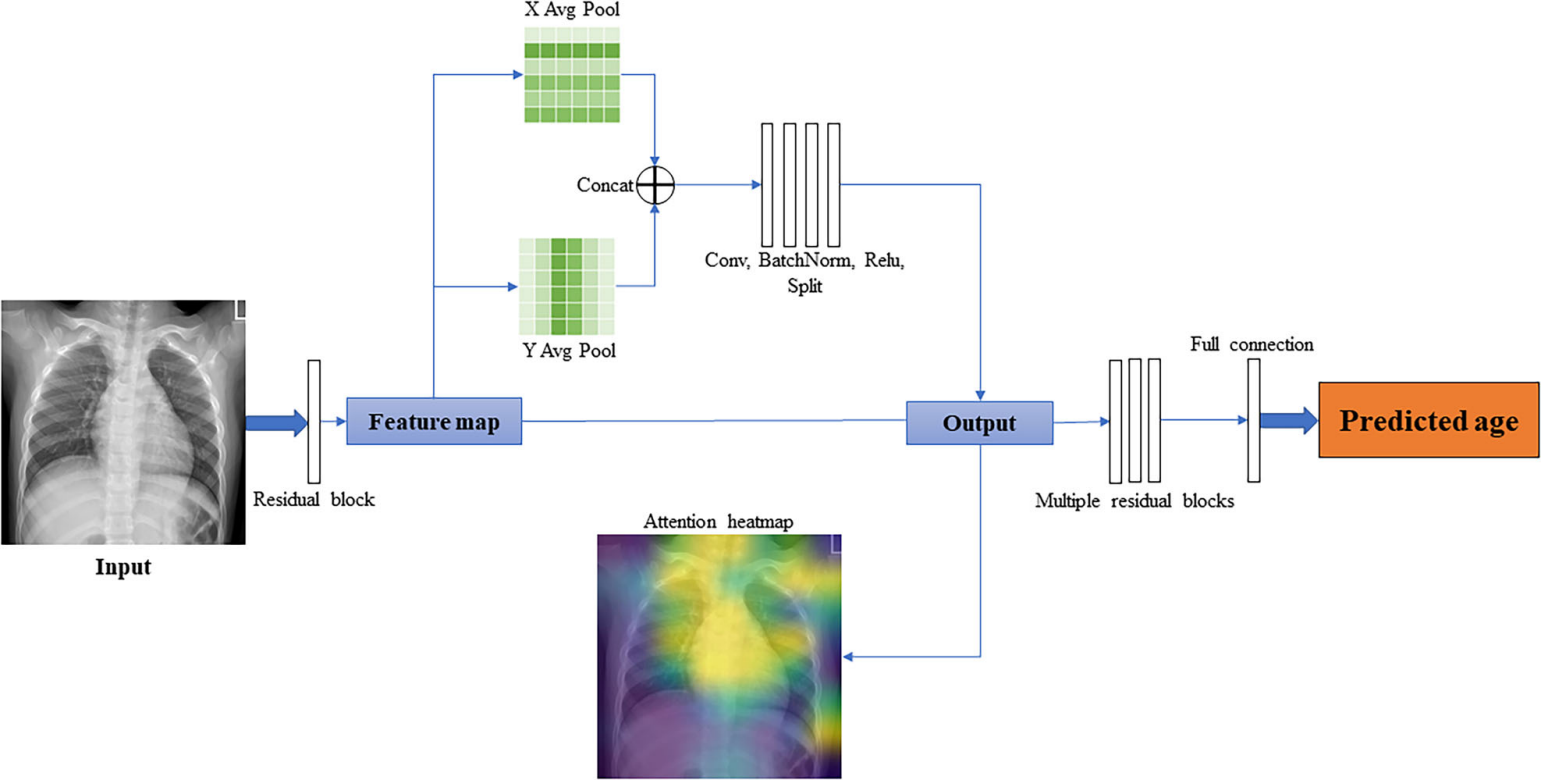
Model structure with coordinate attention mechanism

In this study, we employed a modified ResNet-18 [27] deep neural network as the backbone, integrated with the Coordinate Attention mechanism [28] for the regression task. ResNet is a widely used backbone network in image processing, known for its robust fitting capabilities. The Coordinate attention module was integrated after the residual block to enhance the model’s ability to capture long-range spatial dependencies across both *x*- and *y*-axes. As illustrated in the flowchart of our model in Fig. 2, the outputs of the attention module were subsequently analyzed with additional layers, including extra residual blocks and fully connected layers to generate the predicted age. This integration allowed the model to autonomously identify the most important area of the X-ray image related to age estimation, thereby enabling it to focus on these areas without needing any prior knowledge about this domain.

In summary, our model is capable of accepting chest X-rays as an image input, with the output being the predicted age, without requiring any medical knowledge or additional labeled data.

The application of this model includes two phases: training and validation. During the training phase, the images from the training set were fed to the neural network. These outputs were then compared to the true age to compute the loss function, followed by backpropagation to update the weights of the neural network. In the validation phase, images from both internal and external validation sets were input into the neural network, and the predicted ages were compared to the true values to calculate evaluation metrics. Additionally, during forward propagation, we extracted the output of the coordinate attention layer in the ResNet-18 model. These outputs were then processed to generate attention heatmaps, which visualize the spatial focus of the network by indicating regions of high attention response. The workflow and process of generating heatmaps are shown in Fig. 2.

All chest X-ray images were resized to 256 × 256 pixels before feeding into the neural network. Intensity normalization was not applied. Given the large size of the dataset, no data augmentation techniques were used during training. As for the training hyperparameters, we employed the Adam optimizer with a weight decay of 0.001 and an initial learning rate of 0.0001. Training batch size was 128. The model was trained for 1000 epochs, with the learning rate halved every 200 epochs. For evaluation, the model weights from the final epoch of training were used directly for both internal and external validation. All source code was written in Python, and the deep learning model was implemented using PyTorch 1.12.0. The code is available on GitHub at: https://github.com/bpsl/ageprediction.

### Evaluation metrics

We used Mean Absolute Error (MAE) as the primary evaluation metric for this regression task. However, relying solely on MAE does not provide a comprehensive assessment, as the same MAE value can indicate varying prediction performance across different age groups. For instance, an MAE of 6 months has entirely different implications for a 1-year-old infant compared to a 16-year-old adolescent. To address this limitation, we included the Mean Absolute Percentage Error (MAPE) alongside MAE as additional evaluation metrics to provide a relative measure of error. Furthermore, we divided the validation sets into five age groups to better understand the significance of prediction error across different age ranges: under 1 year, 1–3 years, 3–6 years, 6–10 years, and 10–16 years.

$${MAE}=\frac{1}{n}{\sum}_{i=1}^{n}\left|{y}_{i}-\hat{{y}_{i}}\right|$$

$${MAPE}=\frac{1}{n}{\sum}_{i=1}^{n}\left|\frac{{y}_{i}-\hat{{y}_{i}}}{{y}_{i}}\right|\times 100 \%$$

In addition, we employed Spearman’s rank correlation test for each gender group to further validate the correlation between true and predicted age. The Spearman’s rank correlation coefficient can be defined as:

$${r}_{s}=1-\frac{6\mathop{\sum }_{i=1}^{n}{d}_{i}^{2}}{n\left({n}^{2}-1\right)}$$

Where ${{{{\rm{d}}}}}_{{{{\rm{i}}}}}$ is the difference between the ranks of corresponding predicted age and true age for each sample in the validation sets.

We also generated scatter plots with the actual chronological age on the *x*-axis and the predicted age on the *y*-axis to provide a clearer visualization of the model’s predictions of each case in the validation sets. Additionally, attention heatmaps were applied for each gender and age group to highlight the specific regions on the X-rays that the deep learning model pays more attention to during data processing. All plots were generated using Matplotlib, and Spearman’s test was implemented with SciPy in Python.

## Results

### Data distribution

**Fig. 3:**
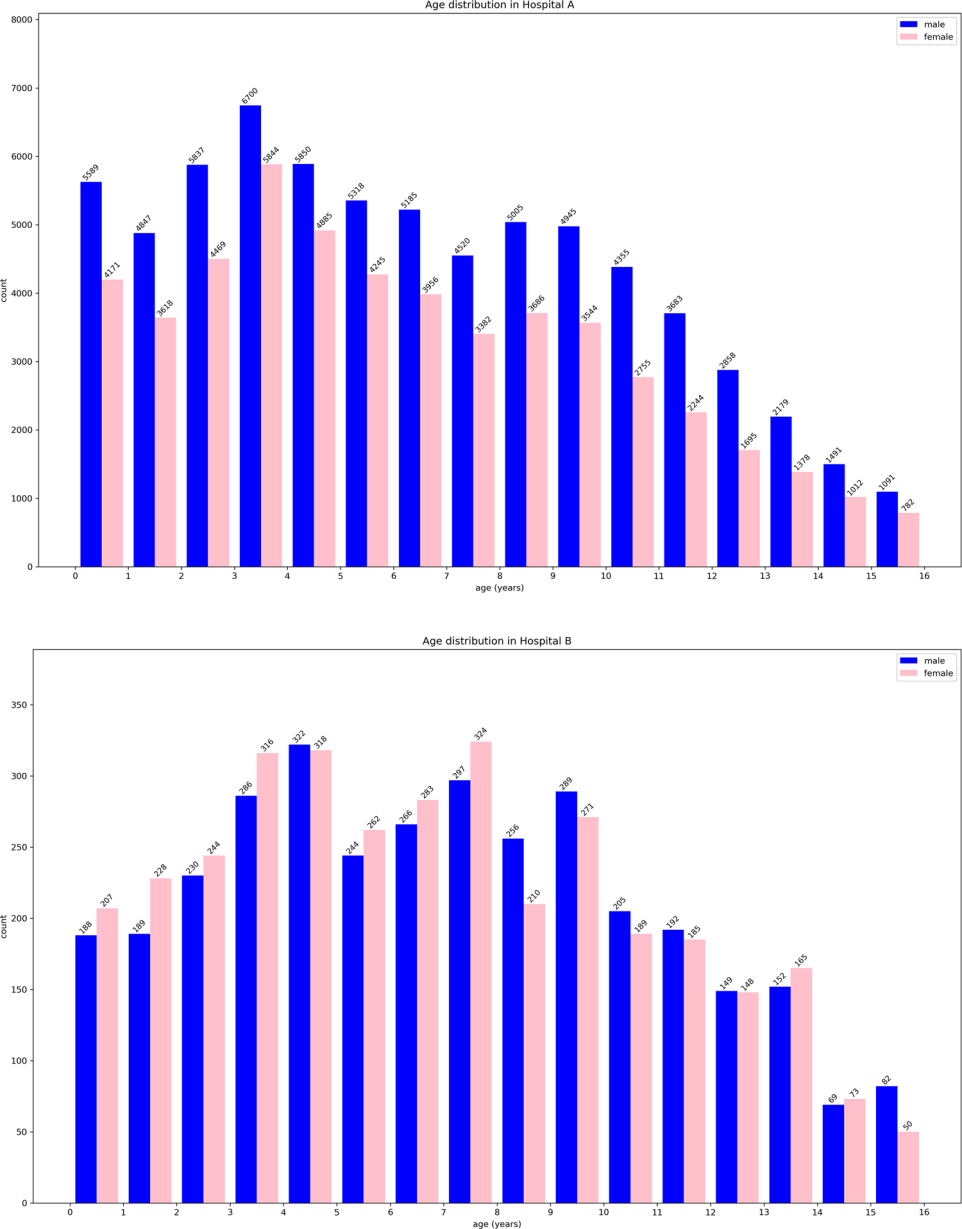
Age (in years) and gender distribution of datasets from two hospitals

We collected a total of 121,119 cases from Hospital A and 6889 cases from Hospital B. The age and gender distributions for these datasets are shown in Fig. 3. Due to the different specialties of the two hospitals, the dataset from Hospital A predominantly comprised younger children, while the dataset from Hospital B had a larger proportion of children in the middle age group. Both hospitals had relatively fewer older children in their datasets. In terms of gender distribution, Hospital A had a higher proportion of boys compared to girls, whereas Hospital B showed a roughly balanced gender ratio.

### Quantitative results

On the internal validation set, the male and female groups achieved an MAE of 5.86 and 5.80 months, respectively, demonstrating strong regression performance. On the external validation set, the MAE was 7.40 months for males and 7.29 months for females. Although the MAE scores of the external validation set were slightly less accurate than those of the internal validation set, they still maintained an acceptable level, demonstrating the model’s robust generalization capacity.

The Spearman rank correlation test revealed a Spearman correlation coefficient of 0.988 (*p* < 0.001) and 0.989 (*p* < 0.001) for the male and female groups respectively on the internal validation set, and 0.982 (*p* < 0.001) for both gender groups on the external validation set, proving a strong positive correlation between the predicted and actual age.

**Fig. 4:**
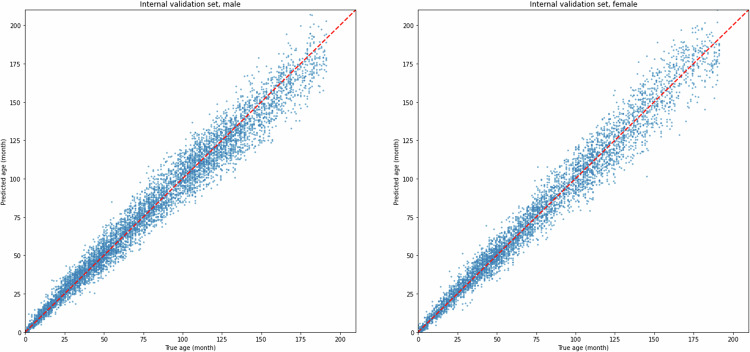
Experiment results on internal validation sets

**Fig. 5:**
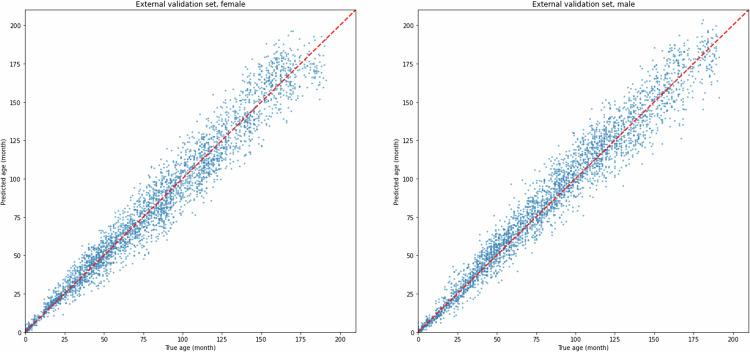
Experiment results on external validation sets. Scatter plots of the model’s prediction results for both the internal and external validation sets. Each point represents an individual sample, with *x*-axis representing true age (in months) and *y*-axis representing predicted age. Points that are closer to the line of identity (*y* = *x*) indicate more accurate age predictions for those cases

Scatter plots in Figs. 4 and 5 provide a visual demonstration of the experiment result on the internal and external validation sets. It is evident that across both datasets and both gender groups, the results of our model closely align with the *y* = *x* line, indicating a strong fitting capability and high accuracy of our model.

### Subgroup analysis

Table 1 presents the quantitative results across five age groups. Since the group of 0–1 year and ‘overall’ category include individuals aged 0 months, we excluded these groups from the MAPE calculation. It can be observed that our model demonstrated favorable performance across all age and gender subgroups, with low MAE and MAPE values. For infants aged under 1 year of age, the prediction error was around 1.3 months, while for children older than 6 years, the mean percentage error remained below 10%.

**Table 1:** Summary statistics and MAE/MAPE for different age groups

		Internal validation set		External validation set	
Age group (Y)		Male	Female	Male	Female
0–1	MAE (m)	1.30 ± 1.46	1.26 ± 1.27	1.33 ± 1.39	1.32 ± 1.34
	MAPE (%)	NA*	NA*	NA*	NA*
1–3	MAE (m)	3.35 ± 2.81	3.34 ± 2.96	3.68 ± 3.41	3.48 ± 3.08
	MAPE (%)	14.4 ± 12.7	14.5 ± 13.0	15.8 ± 14.8	15.0 ± 13.3
3–6	MAE (m)	4.97 ± 4.02	4.63 ± 3.67	6.45 ± 5.13	5.78 ± 4.63
	MAPE (%)	9.6 ± 7.8	9.1 ± 7.4	13.0 ± 9.8	11.2 ± 9.1
6–10	MAE (m)	6.97 ± 5.29	7.28 ± 5.47	8.46 ± 6.81	8.90 ± 6.65
	MAPE (%)	7.0 ± 5.4	7.3 ± 5.5	8.6 ± 7.0	9.2 ± 7.0
10–16	MAE (m)	9.63 ± 7.21	10.11 ± 7.33	10.32 ± 7.54	10.67 ± 7.62
	MAPE (%)	6.3 ± 4.7	6.5 ± 4.8	6.7 ± 4.9	7.0 ± 5.0
Overall	MAE (m)	5.86 ± 5.39	5.80 ± 5.41	7.40 ± 6.56	7.29 ± 6.40
	MAPE (%)	NA*	NA*	NA*	NA*
	AEQs	4.31 (1.74, 8.48)	4.26 (1.70, 8.32)	5.41 (2.26, 10.77)	5.45 (2.10, 10.61)
	APEQs	7.18 (3.24, 13.22)	7.27 (3.35, 13.22)	8.13 (3.68, 16.65)	8.11 (3.77, 14.65)

Values are presented as mean ± standard deviation or median (25th percentile, 75th percentile)

*MAE* mean absolute error, *MAPE* mean absolute percentage error, *Y* years, *m* months, *AEQs* absolute error quartiles, *APEQs* absolute percentage error quartiles

MAPE is not available for the ‘0–1 year’ and ‘overall’ groups because the presence of 0-month cases in these groups results in division by zero in MAPE calculation.

Performance variability was observed across age groups, with MAE generally increasing but MAPE decreasing as age progressed. This trend is consistent with the inherent properties of these two metrics: as the chronological age increases, the same absolute error accounts for a smaller percentage of age. Therefore, jointly using MAE and MAPE metric provides a more balanced interpretation of the model’s real performance. This trend with age is also observed in the scatter plots (Fig. 5).

The internal validation set showed slightly better results than the external validation set across all age groups; however, the gap is still within an acceptable range. This highlights the robust generalization ability of our model while also suggesting potential room for improvement when applied to unseen data.

### Heatmap analysis

**Fig. 6:**
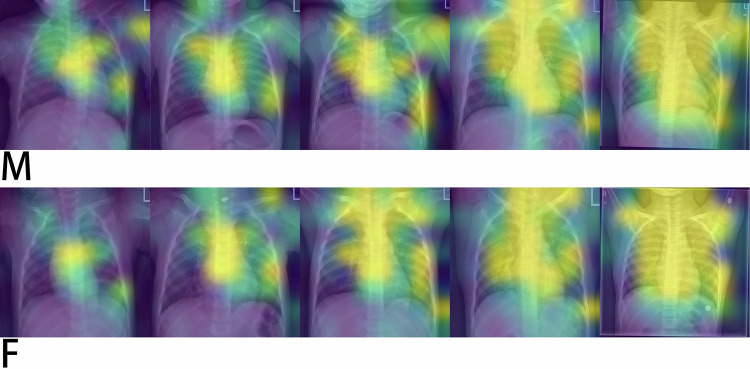
Attention heatmaps of sample case images on different age and gender groups: 0–1, 1–3, 3–6, 6–10, 10–16 years. M: Male. F: Female. The brighter areas indicate regions where the deep learning model pays more attention

This section presents attention heatmaps in each gender and age group in Fig. 6. The brighter areas on these images indicate regions where the deep learning model “pays more attention.” It can be observed that the deep learning model’s primary focus was around the heart and mediastinum, likely due to the high concentration of relevant information in these areas, including the spine, mediastinum, heart, and great vessels, which are central to evaluating both the skeletal and soft tissue maturation. The graphs also show that the deep learning model also focused somewhat on the neck and shoulder regions, as well as a smaller portion of the costophrenic angles, particularly on the left side. This indicates that the model attempted to utilize the information from the growth plates of the bones and joints in these regions. In contrast, there was lower activation in the subdiaphragmatic region and within the lungs, proving these regions are less important for age estimation.

A general trend is that with increasing age, the level of overall quantitative attention activation increased, represented by larger and more pronounced bright regions on the heatmap. However, the characteristics of the activated regions remained similar across age groups. Additionally, there was no significant difference between the two gender groups.

## Discussion

Predicting children’s age is a valuable task in medical practice, relevant to both forensic investigations and clinical assessments of pediatric growth and development. Evaluating children’s growth and development is crucial for early detection of growth disorders, with radiology playing a vital role in this scenario. Given that chest X-ray is now the most commonly used technique in pediatric radiological examinations [23], our approach explores the potential of using chest X-rays to predict a child’s age. If this “chest age” model shows high prediction accuracy in experiments, clinical applications based on this approach could reduce the need for additional imaging and consequently minimize radiation exposure.

In this study, we built a large dataset of chest X-rays from over 120,000 Chinese children, and successfully trained a deep learning model on it. High accuracy level was proved on both internal and external validation sets across all gender and age groups. On the internal validation set, our model achieved an overall MAE of less than 6 months, while on the external validation set, the overall MAE was around 7 months. A Spearman rank correlation test confirmed a very strong positive relationship between predicted and true age, with a Spearman correlation coefficient greater than 0.98 in both internal and external validation sets. Notably, this study is among the few that comprehensively evaluated an age prediction model across different age groups in children: for infants under 1 year of age, the model achieved a MAE of as low as around 1.3 months, while the MAPE remained below 10% in children over 6 years old, indicating high accuracy level for minors across all age ranges.

Enhanced with Coordinate Attention mechanism that analyzes images holistically, our model autonomously identified areas requiring additional attention without needing any prior knowledge about chest X-rays or any labels. Attention heatmaps further demonstrate that our model can integrate information from different anatomical structures and simultaneously focus on multiple regions of interest, including the heart, mediastinum, and bones that contribute to age assessment, to perform a comprehensive analysis.

Some existing studies [21, 25, 26] have explored the application of chest X-rays to estimate age in general populations, primarily on adults, with applications in forensic science. In contrast, our model specifically targets children, as age prediction holds potential for assessing growth and development, with important clinical implications. In this context, compared to some other studies that tried to predict pediatric age with deep learning, our study employs a larger dataset, surpassing even most of the existing wrist bone age studies [15]. This extensive dataset allows for more robust deep learning training, thereby enhancing predictive accuracy. Although the accuracy of our model slightly falls short of the best wrist radiograph-based models [24], it has performed a level comparable to mainstream wrist radiograph-based models [13, 29–31], and have surpassed nearly all other studies that predict pediatric age with alternative radiological modalities, such as those with abdominal CT scouts and knee joint X-ray [20, 21], which typically achieved a MAE of around 1 year or more.

This research holds significant potential value. First, minimizing unnecessary radiation exposure is a crucial concern in pediatric radiology [22]. Given that chest X-ray is the most common radiological examination in pediatrics [23, 32], by utilizing the existing images with deep learning to evaluate growth and development, we can reduce the need for additional wrist X-rays. Second, since the development of different body regions occurs asynchronously [32], our model offers an alternative perspective by focusing on the chest region. Even in cases where wrist radiographs are indispensable, our model can serve as a valuable tool and provide a secondary reference. Lastly, radiological age assessment criteria vary across different regions, ethnic groups, and even over time, as they reflect the unique growth patterns of each population. Our model, leveraging deep learning-based automatic analysis and large chest X-ray datasets, offers a rich resource for developing more flexible age prediction tools. By training deep learning models on extensive datasets from various populations, these models can quickly adapt to reflect the specific characteristics of the group being studied.

This study has some shortcomings that are currently hard to solve. First, although we have tried to exclude children diagnosed with growth abnormalities from the dataset to the greatest extent possible, some cases could not be completely excluded. This is because some children may have an undiagnosed growth disorder, or have sought medical care at other institutions but have not left a record in our system. Second, although our model has achieved a relatively high level of accuracy, there is still a gap between our model and the current best-performing models based on wrist radiographs, with some literature reporting an MAE as low as around 0.4 years. However, while many wrist bone age datasets have been extensively studied and optimized, our dataset has not yet undergone full algorithmic exploration. This indicates potential for further accuracy improvements through opening dataset and more algorithmic optimization.

Certain future work remains to be done based on our current findings. As a pioneering study in this field, our research primarily included healthy children and confirmed the potential value of chest X-rays in assessing pediatric age. However, the clinical implications of the implemented “chest age” require further investigation. Future studies will focus on examining the diagnostic consistency between our proposed “chest age,” wrist bone age and chronological age, and assess the diagnostic performance of our proposed model in pediatric growth disorders. If age prediction with chest X-ray is shown to be clinically meaningful, this deep learning-based approach will also be worth exploring across other imaging modalities, such as other X-rays, CT, and MRI, to unlock further potential of deep learning in existing radiological techniques.

In conclusion, this study demonstrates the feasibility of using chest X-rays combined with a deep learning model to predict pediatric age. By exploring a large original dataset with Coordinate Attention enhanced ResNet model, our approach achieved promising accuracy levels on both internal and external validation sets, with a high MAE score that nears reliable established bone age prediction models based on wrist X-rays.

The findings indicate that chest X-rays hold valuable diagnostic information for age assessment, offering a complementary method to conventional radiographic techniques.

## Data Availability

Source code is available on GitHub at https://github.com/bpsl/ageprediction. The original images are available upon reasonable request from the corresponding author.

## Abbreviations

CT: Computed tomography
MAE: Mean absolute error
MAPE: Mean absolute percentage error
MRI: Magnetic resonance imaging
